# Functional Assessment of Microplasma-Sprayed Hydroxyapatite-Zirconium Bilayer Coatings: Mechanical and Biological Perspectives

**DOI:** 10.3390/ma18143405

**Published:** 2025-07-21

**Authors:** Sergii Voinarovych, Serhiy Maksimov, Sergii Kaliuzhnyi, Oleksandr Kyslytsia, Yuliya Safarova (Yantsen), Darya Alontseva

**Affiliations:** 1 E.O. Paton Electric Welding Institute, National Academy of Sciences of Ukraine, 11 Kazymyr Malevych Str., 03150 Kyiv, Ukraine; serge.voy@gmail.com (S.V.); maksimov@paton.kiev.ua (S.M.); serg3319@ukr.net (S.K.); kisl@i.ua (O.K.); 2 LLC Paton Innovations, Kyiv 03150, Ukraine; 3 Laboratory of Bioengineering and Regenerative Medicine, Center for Life Sciences, National Laboratory Astana, Nazarbayev University Kazakhstan, Astana 010000, Kazakhstan; 4 School of Digital Technologies and Artificial Intelligence, D. Serikbayev East Kazakhstan Technical University, 19 Serikbayev Street, Ust-Kamenogorsk 070010, Kazakhstan

**Keywords:** titanium implants, hydroxyapatite (HA), zirconium (Zr), microplasma spraying (MPS), coatings, adhesive strength, roughness, biocompatibility

## Abstract

Hydroxyapatite (HA) has become a widely used material for bone grafting and surface modification of titanium-based orthopedic implants due to its excellent biocompatibility. Among various coating techniques, microplasma spraying (MPS) has gained significant industrial relevance. However, the clinical success of HA coatings also depends on their adhesion to the implant substrate. Achieving durable fixation and reliable biological integration of orthopedic implants remains a major challenge due to insufficient coating adhesion and limited osseointegration. This study addresses challenges in dental and orthopedic implantology by evaluating the microstructure, mechanical properties, and biological behavior of bilayer coatings composed of a zirconium (Zr) sublayer and an HA top layer, applied via MPS onto titanium alloy. Surface roughness, porosity, and adhesion were characterized, and pull-off and shear tests were used to assess mechanical performance. In vitro biocompatibility was tested using rat mesenchymal stem cells (MSCs) to model osteointegration. The results showed that the MPS-fabricated Zr–HA bilayer coatings achieved a pull-off strength of 28.0 ± 4.2 MPa and a shear strength of 32.3 ± 3.2 MPa, exceeding standard requirements. Biologically, the HA top layer promoted a 45% increase in MSC proliferation over three days compared to the uncoated titanium substrate. Antibacterial testing also revealed suppression of *E. coli* growth after 14 h. These findings support the potential of MPS-applied Zr-HA coatings to enhance both the mechanical integrity and biological performance of titanium-based orthopedic implants.

## 1. Introduction

Pure titanium (Ti) and titanium alloys are widely used in orthopedics for the manufacture of implants due to their advantages, such as their mechanical properties and high corrosion resistance. However, despite these characteristics of Ti alloys, implants made of them do not always provide the necessary osseointegration. The most general statistical impact of Ti and Ti alloy toxicity is reflected in [1] where, based on an analysis of the literature from 1991 to 2018, its negative impact was mentioned with partial or prolonged contact with the human body environment at different implantation sites. Statistics showed 1378 sources containing data on the toxicity of titanium and its alloys in the description. About 900 sources are related to hypersensitivity and corrosion, and 256 sources on “yellow nail” syndrome with the manifestation of concomitant diseases associated with its presence in the body. From the provided statistical data, it follows that titanium, as a biocompatible material, is significantly overestimated [2] and the conclusions made by some authors indicate a further increase in cases of its negative impact on the human body in the future [3,4]. However, the negative impact is reduced by the application of coatings from metals, ceramics, carbon, and their combinations [5,6,7,8].

The Young’s modulus of commercially pure titanium (cp-Ti) is three times higher than that of human cortical bone, which leads to stress shielding, causing resorption of the bone tissue surrounding the implant and weakening of its fixation. It is known that porosity in coatings on endoprostheses is considered not only to promote bone ingrowth and ensure adhesion of the implant to the bone but also to ensure alignment of the elastic modulus of the coating with the elastic modulus of the bone [9]. Therefore, the use of porous coatings not only promotes biological fixation of the implant in the bone through bone ingrowth into the pores of the coating but also reduces the “stress shielding” effect. There are active studies on the formation of porous surface structures in the manufacture of endoprostheses; in particular, the application of metallic porous coatings [10].

Recently, zirconium (Zr) and its alloys have been considered as an alternative material for implants, since they have a number of advantages over titanium alloys: firstly, the technological methods for obtaining zirconium provide a higher purity of the material; secondly, in air zirconium compares favorably with titanium in that it does not absorb hydrogen, which leads to embrittlement at temperatures above 50–70 °C during technological processing. Zr is also inert in air at temperatures up to 300 °C due to the presence of a protective oxide film on its surface. Zr and its alloys are characterized by high corrosion resistance in organic compounds (higher indicators than such stable metals as titanium and niobium), biological inertness, and low ability to interact with various aggressive environments [11]. Zr and its alloys are known for their lower rejection rates compared to Ti-based alloys, and zirconium’s surface inhibits bacterial adhesion better than titanium [12,13].

However, most exogenous materials introduced into biological tissue cause fibrous encapsulation. Fibrous encapsulation is a protective measure of the body, but, unfortunately, it also prevents strong and long-term osseointegration of the implant with the bone, which subsequently leads to loosening and loss of the implant. For this reason, wide attention has been drawn to improving the biocompatibility of the surface of implants [14].

Hydroxyapatite (Ca_10_(PO_4_)_6_(OH)_2_) is one of the most attractive materials for implantation into human bone tissue due to its excellent biocompatibility and osteoconductivity, and hydroxyapatite (HA) coatings have been shown to be effective in promoting osseointegration and in enhancing implant stability and bone formation [15,16,17,18]. 

The literature provides a fairly large list of methods for forming HA coatings, including sol–gel, plasma spray, pulsed deposition, electrophoretic deposition, biomimetics, microarc oxidation, RF sputtering, microwave-assisted synthesis, laser-induced single-step coating, electrostatic spray-assisted vapor deposition, electrospinning, spray pyrolysis, pulser-laser deposition, drop casting, and the combination of two or more coating methods [19,20,21].

However, the most common method for forming HA coatings is plasma spraying due to its relatively low cost and ability to coat complex-shaped areas. In addition, plasma spraying is the only process that is actually approved by the U.S. Food and Drug Administration (FDA) for use in applying HA coatings to medical implants [22,23].

Recently, work has been carried out to develop the technology of microplasma-sprayed coating of medical parts. The technology of microplasma spraying (MPS) of coatings makes it possible to apply coatings using both powder and wire materials on substrates made of various materials. MPS enables the creation of porous coatings on metallic endoprostheses using various biocompatible materials, including Ti, tantalum (Ta), zirconium (Zr), and calcium phosphate-based ceramics such as HA. This is particularly valuable for fabricating metal–ceramic bilayer coatings. Magnesium alloys are promising candidates for biodegradable implants; however, their rapid degradation in physiological conditions requires surface treatment techniques—such as plasma electrolytic oxidation coatings doped with cerium oxide (CeO_2_)—to improve their corrosion resistance and biocompatibility [24]. When coating small components like implants, MPS offers a much higher material utilization rate compared to conventional atmospheric plasma spraying. This advantage stems from the smaller spray spot diameter, typically between 3 mm and 5 mm. Furthermore, MPS generates minimal thermal load on the substrate due to the low power of the microplasmatron, making it well suited for coating thin-walled or small parts without causing overheating or deformation [25,26,27].

One of the critical factors ensuring successful fixation of the implanted product is the adhesion strength of the biocompatible coating to the implant surface. Poor adhesion at the implant–coating interface raises concerns about its long-term functioning in the patient’s body. Therefore, improving the bond strength between the implant surface and the biocompatible coating is a general requirement regardless of the coating application methods used. To improve the adhesion of the coating to the substrate during plasma spraying, gas abrasive treatment is traditionally used to roughen the metal surface, but sometimes this is not enough to achieve positive results [28].

To meet the requirements of long-term use of endoprostheses, further research is needed to improve the bond strength between the microplasma-sprayed HA coating and the implant, including the use of bonding sublayers with the required parameters of porosity and surface roughness. At the same time, the coatings used must also be studied for biocompatibility to prevent an adverse biological reaction of the implant surface, which can lead to poor integration of titanium implants with the surrounding tissue. This is particularly important for osseointegration, where effective biological interaction between the implant surface and adjacent bone tissue is critical to implant success. Enhancing this interaction promotes greater biocompatibility and ultimately leads to better clinical outcomes in orthopedic, dental, and other implant-related procedures [29].

The aim of this study was to evaluate the roughness and porosity of biocompatible metal-ceramic two-layer coatings made of HA and a Zr sublayer applied by microplasma spraying, determine the value of their adhesion strength for normal tear and shear, and study the biocompatible and bacterial resistance properties of the obtained coatings.

The ultimate goal is to provide scientifically validated recommendations for selecting MPS parameters to enhance the bioactivity of endoprosthetic implants. Additionally, this work seeks to identify the influence of MPS parameters on the microstructure and properties of Zr–HA coatings, laying the foundation for future advancements in the field of dental and orthopedic implant technology.

## 2. Materials and Methods

Solid Zr wire with a diameter of 0.3 mm was used as the material for spraying. The chemical composition of the wire is given in Table 1.

**Figure 1:**
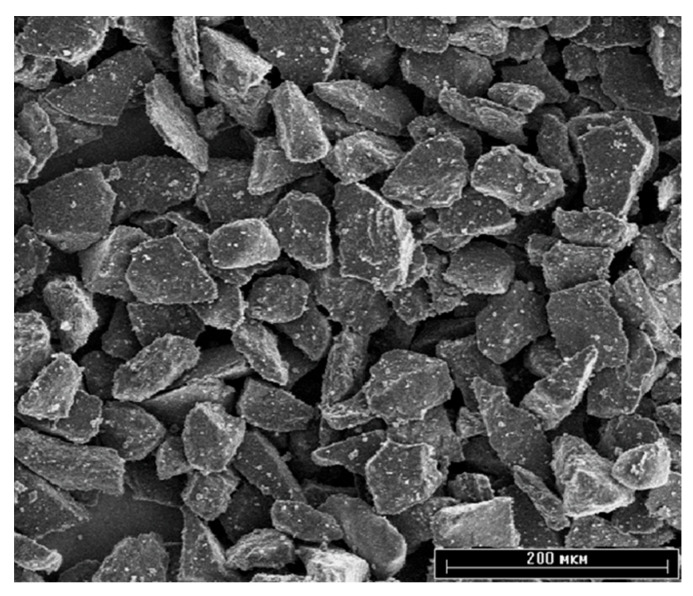
SEM image of HA raw powder. Scale bar is 200 µm.

Ceramic hydroxyapatite powder (Center for Scientific and Technical Solutions “BIOMATTEKH”, Kyiv, Ukraine) of high purity (>99%) with a particle size of 40–63 µm was used as the material for the top layer of the coating (Figure 1).

The coatings were applied using a microplasma spraying (MPS) unit using an MPS-004 microplasmatron manufactured by the E.O. Paton Electric Welding Institute (Kyiv, Ukraine) [31]. The features of the microplasma spraying process of biocompatible coatings have been described in more detail in previous work [32]. Rolled Ti6V4Al (Grade 5), Table 2, was used as the sample material.

Before spraying, the end surface of the samples was degreased and gas-abrasive treated, the mode of which is presented in Table 3. Normal electrocorundum 25A F30 (500–800 μm) was used as an abrasive (ISO 6344-2:1998 [34]). After gas-abrasive treatment, the samples for coating application were subjected to ultrasonic surface cleaning in an isopropyl alcohol environment for 15 min. The coatings were applied to the surfaces of the samples no more than two hours after the above preparation. Plates measuring 10 × 15 mm with a thickness of 2.5 mm were used for metallographic and mechanical analyses, while discs with a diameter of 10 mm and thickness of 1 mm were used for biological assessments.

The porous Zr coating was applied by the microplasma spraying method with the following process parameters: current strength I = 16 A; plasma gas flow rate Qpl = 2.7 slpm; spraying distance H = 0.04 m; and wire feed rate Vw = 2.9 m/min. Then, to form a two-layer Zr–HA coating, a hydroxyapatite coating was applied on top with the following process parameters: current strength I = 35 A; plasma gas flow rate Qpl = 2 slpm; and spraying distance H = 0.1 m

### 2.1. Surface Roughness Assessment

#### 3D Laser Scanning

Surface roughness was evaluated using two complementary techniques. A Keyence VK-X1000 confocal laser scanning microscope (Neu-Isenburg, Germany) was employed to measure the surface roughness parameters Sa and Sq in accordance with ISO 25178-600 [35]. The Sa value represents the average deviation of surface points from the mean plane (mean roughness), while Sq corresponds to the root mean square of the surface height distribution, reflecting the standard deviation. Area-based roughness metrics like Sa and Sq are considered more informative than line-based parameters for characterizing surface topography. Measurements were conducted over an area of approximately 700 × 500 μm^2^, and for each sample, three randomly selected regions were analyzed to ensure reproducibility. In addition, surface roughness was assessed using a contact roughness and waviness tester SSR300 (Hangzhou Mituolinke Technology Co., Ltd., Hangzhou, China), following the relevant standard protocols [36].

The surface topography and chemical composition of the coatings was studied using scanning electron microscopes JSM-6060 (JEOL, Tokyo, Japan) and JSM-6390LV (JEOL, Tokyo, Japan) equipped with an EDX INCA ENERGY attachment (Oxford Instruments, Oxford, UK).

The adhesion strength of the coatings to the base under normal peeling was determined according to the ASTM F1147 [37] method, and the adhesion strength of the coatings to the base under shear was tested according to the ASTM F1044 [38] method. In both methods, the adhesion strength is measured by peeling the coating from the base through a counter sample glued to the outer surface of the coating using VK-9 glue (Khimprom, Ukraine)—65% epoxy resin and 35% hardener. Gluing was carried out with a compression force of 50 kPa until the glue completely hardened, which occurred within 5–6 h when heated to 70 °C. After the resin had hardened, the glued samples were mounted in a fixture for the appropriate tests.

**Figure 2:**
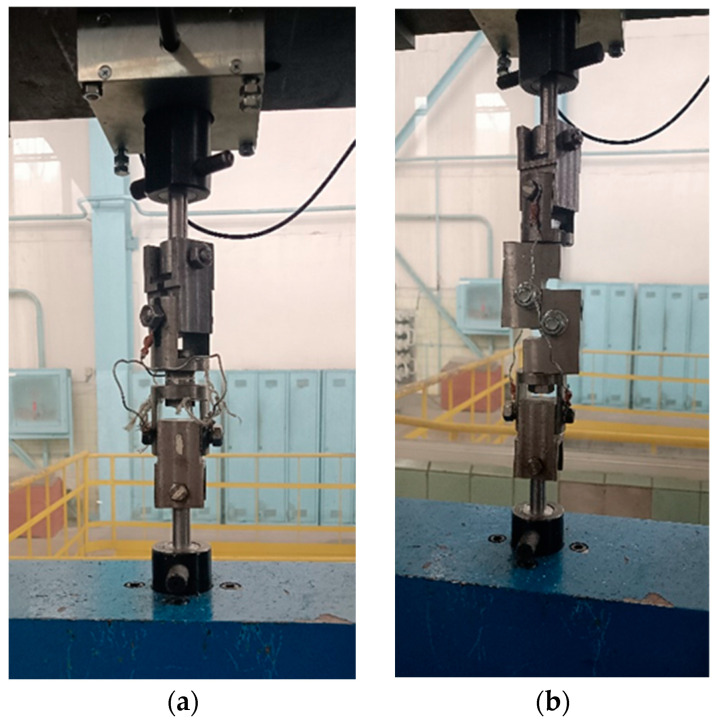
Equipment for testing the adhesion strength of coatings: (**a**) peel; (**b**) shear.

Tensile and shear testing of each set of glued sample assemblies were performed on a universal mechanical machine 2054 P-5 at the same load rate of 2 mm/min. To fix the glued sample assemblies with a coating in the tensile testing machine, a rig with a hinged joint was used (Figure 2).

After the connection was broken, the load value was recorded and the nature of the destruction zone (coating-base, coating-adhesive, and destruction inside the coating) was visually assessed.

### 2.2. CCK8 Proliferation Assay

To assess the in vitro proliferation of rat mesenchymal stem cells (MSCs), a CCK-8 assay (Sigma Aldrich, Cat. No. 96992, St. Louis, MO, USA) was employed. MSCs (5 × 10^3^ cells per well) were seeded in a 96-well plate and cultured for 72 h in a medium containing implant extracts. Following the incubation period, 10 µL of the CCK-8 reagent was added to each well, and the plates were further incubated for 4 h. Absorbance was then recorded at 450 nm using a BioTek Synergy H1 microplate reader (Winooski, VT, USA). Cell numbers were calculated based on a standard calibration curve. As a control, MSCs were cultured in standard DMEM without exposure to implant-derived media.

### 2.3. Alkaline Phosphatase Activity (ALP)

Cells were plated at a density of 8 × 10^4^ per well in 48-well plates and cultured until they reached approximately 90% confluence. Subsequently, they were exposed to an osteogenic differentiation medium that had been pre-incubated with implants for three days. After a two-week incubation period, culture supernatants were harvested, and alkaline phosphatase (ALP) activity was quantified using a commercial assay kit (Abcam, ab83369, Cambridge, UK).

### 2.4. Antibacterial Assay

Antibacterial activity was assessed using Escherichia coli strain HB101/K12 (Bio-Rad, 1660408, Hercules, CA, USA). Cultures were incubated at 37 °C for 24 h in a BioTek H5 microplate reader (BioTek, Hercules, CA, USA), with the optical density at 600 nm (OD600) recorded hourly.

### 2.5. SEM Preparation

Rat bone marrow-derived mesenchymal stem cells (BM-MSCs) were seeded onto titanium discs, both with and without hydroxyapatite (HA) coating, at a density of 1 × 10^4^ cells per disc. The cells were cultured in complete DMEM for 48 h. Following incubation, the cells were fixed with 2% paraformaldehyde for 10 min, then stained with 1M osmium tetroxide for 1 h. Subsequently, samples were rinsed twice in Milli-Q water (10 min per wash), dehydrated through a graded ethanol series (70% to 100%), and treated with hexamethyldisilazane (HDMS) for 30 min. Prior to imaging, all samples were sputter-coated with a 20 nm gold layer for 60 s using a Q150T ES Sputter Coater (Quorum Technologies, London, UK). Surface morphology was examined using a scanning electron microscope (JSM-IT200(LA), JEOL, Tokyo, Japan).

In vitro tests were performed using animal mesenchymal stem cells (MSCs), which are often used to measure biocompatibility characteristics and generally allow for comparison of results obtained in different studies. The choice of MSCs provides a more accurate and dynamic model for in vitro studies to understand how implants interact with human tissues at the cellular level.

## 3. Results

**Figure 3:**
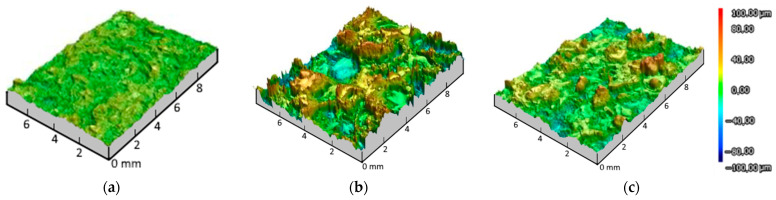
3D laser scanning of Ti6Al4V alloy substrate after gas-abrasive treatment (**a**); Zr coating (**b**); HA coating (**c**).

The results of 3D laser scanning of Ti6Al4V alloy substrate subjected to gas-abrasive treatment and Zr and HA coatings on Ti6Al4V alloy substrate are represented in Figure 3.

Sa parameters for Ti6Al4V appeared to be the lowest at 4.62 ± 0.09 μm when it served as a base for coating deposition. The roughness parameter Sa of the Zr coating showed the roughest surface with Sa equal to 17.03 ± 1.90 μm, while the Sa value of the HA coating was 9.84 ± 0.47 μm.

**Figure 4:**
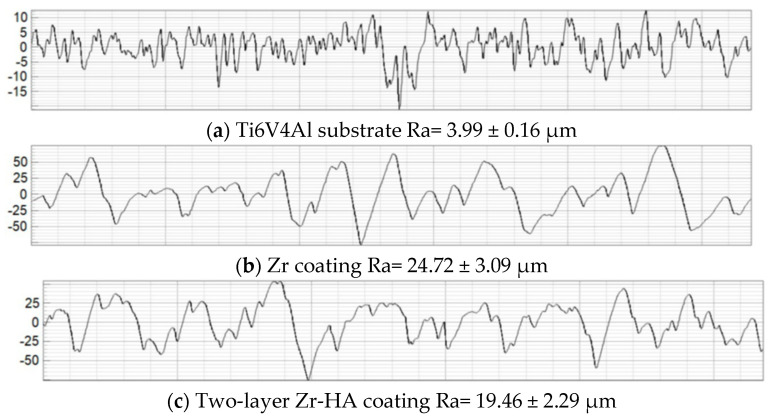
Surface profilograms: (**a**) titanium alloy substrate after gas-abrasive machining; (**b**) Zr coating; (**c**) two-layer Zr–HA coating.

The results obtained in this study allowed us to estimate the average roughness (Ra) of the Ti6V4Al substrate, Zr coating and two-layer Zr–HA coating. For comparison, Figure 4 shows the results of measuring the surface roughness of the titanium alloy substrate subjected to gas-abrasive processing and two types of microplasma-sprayed coatings.

As can be seen in Figure 4, after gas-abrasive treatment, the Ti6V4Al (Grade 5) sample showed the lowest roughness, Ra = 3.99 ± 0.16 μm, while the sprayed Zr coating and the two-layer Zr–HA coating showed an almost 5–6-fold increase in roughness, Ra = 24.72 ± 3.09 μm and Ra = 19.46 ± 2.29 μm, respectively.

**Figure 5:**
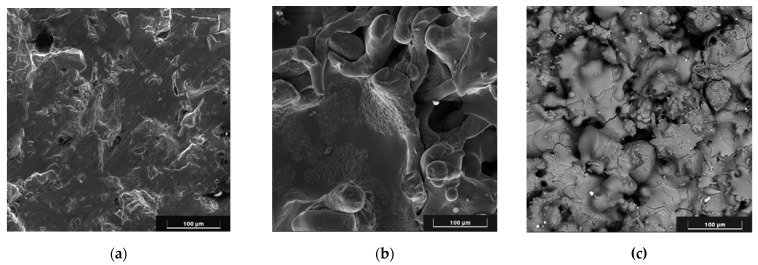
SEM images of the surface: Ti6V4Al substrate after gas-abrasive treatment (**a**); Zr coating (**b**); two-layer Zr–HA coating (**c**). Scale bars are 100 µm.

SEM images of the substrate and coating surfaces are shown in Figure 5.

As can be seen in Figure 5, pores up to 100 μm in size are observed on the surface of the coatings.

**Figure 6:**
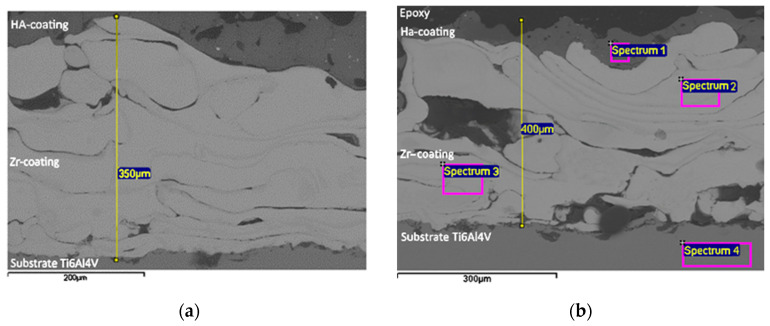
SEM images of Zr–HA coatings: a section with medium pores and an indication of the thickness of the intermediate layer (**a**); a section with large pores, an indication of the thickness of the two-layer coating and with marking of the EDX spectra (**b**). Scale bars are 200 µm (**a**) and 300 µm (**b**).

Furthermore, the cross-section of Zr–HA two-layer coatings were obtained using scanning electron microscopy (SEM) (Figure 6). The chemical composition of the two-layer coating was analyzed with EDX in certain spectra shown in Figure 5 and outlined in Table 4.

The results of the coating microstructure analysis (Figure 6) show the presence of pores with sizes from 150 μm to 50 μm in the Zr coating, while the volume porosity of Zr coatings reached values of up to 20.3%. In the case of the HA coating, the porosity reached 10%, and the pore sizes were up to 50 μm. The results of the EDX analysis (Table 4) confirm the chemical composition of the titanium alloy substrate/Zr–HA coating system.

**Figure 7:**
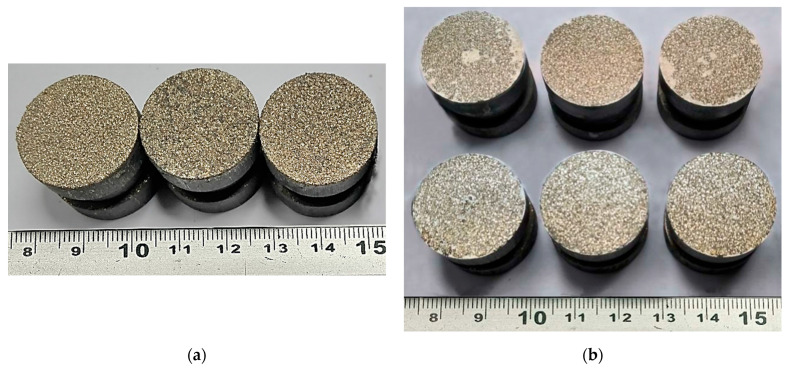
Appearance of samples of sprayed Zr coatings for measuring the adhesion strength under normal peel stress: (**a**) before testing; (**b**) after testing.

**Figure 8:**
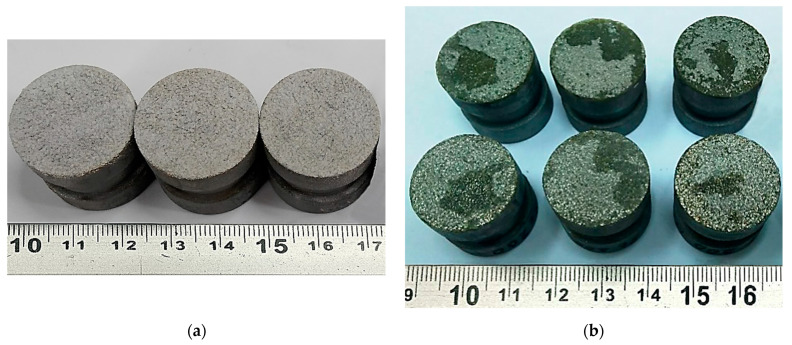
External appearance of samples sprayed with HA coating on a Zr sublayer for measuring the adhesion strength for normal peel: (**a**) before testing; (**b**) after testing.

The study of the adhesion strength of the coatings to the base under normal tearing was carried out using standard cylindrical samples with a diameter of 25 mm and a height of 30 mm (samples shown in Figure 7 and Figure 8).

The average static peel strength of the Zr coating was 27.9 ± 3.8 MPa.

From the analysis of the coating destruction zone, it was established that the rupture occurs between the layers of the applied Zr coating (Figure 7b).

The average static peel strength of the HA coating on the Zr sublayer was 28.0 ± 4.2 MPa. In accordance with the methodology of testing the adhesion strength of coatings to the base in shear, using cylindrical samples (diameter 19.05 mm and length 25.4 mm) (Figure 8), the average static shear strength of the HA coating on the Zr sublayer was determined to be 32.3 ± 3.2 MPa.

**Figure 9:**
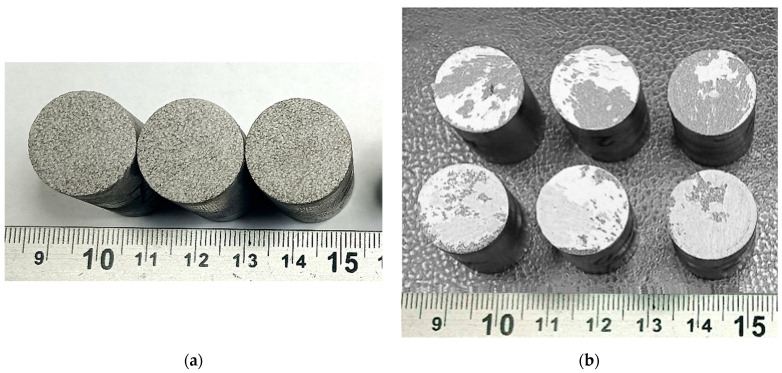
Appearance of samples sprayed with HA coating on a Zr sublayer for measuring shear adhesion strength: (**a**) before testing; (**b**) after testing.

The study of the coating destruction zone showed that the applied HA layer separates from the Zr sublayer, with the residual HA coating of about 60–90% remaining on the sample (Figure 9b).

**Figure 10:**
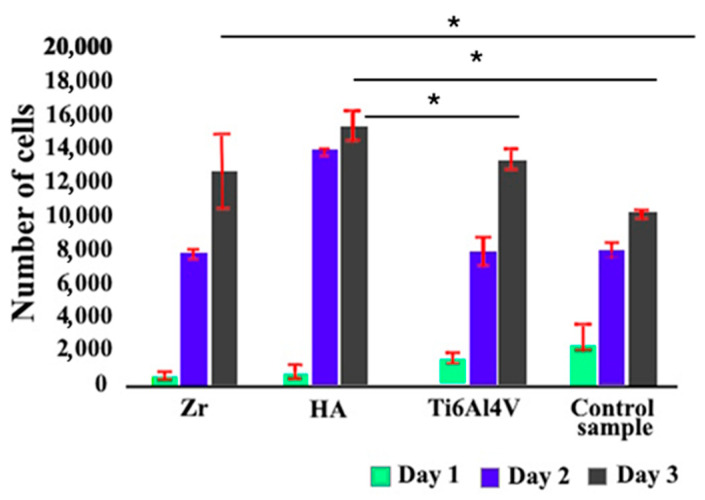
Mesenchymal stem cell proliferation over 72 h assessed by CCK-8 assay. Cells were cultured in an implant-conditioned medium for three days, and proliferation was quantified using the CCK-8 kit (96992, Sigma Aldrich). * *p* ≤ 0.05 indicates statistical significance.

To evaluate the biocompatibility of microplasma-sprayed coatings, cell proliferation was assessed using the Cell Counting Kit-8 (CCK-8) assay. This method, which measures cellular metabolic activity, provides a sensitive and reliable means of quantifying cell viability and proliferation—key indicators of the compatibility and functional performance of implant coatings. Selected findings from these experiments are presented in the referenced studies [27,39]. The supplemented results of the proliferation analysis are presented in Figure 10.

The results of the study showed that the proliferation rate for cells on the surface of Ti6Al4V alloy after gas-abrasive processing, which is widely used as a material for implants, was lower by 23% compared to the control group. Zirconium coating had a minor effect on osteogenesis, increasing osteogenic differentiation by 5% compared to the Ti6Al4V alloy. The study of the proliferation rate for cells on HA coatings showed a statistically significant increase of approximately 45% compared to the control group.

**Figure 11:**
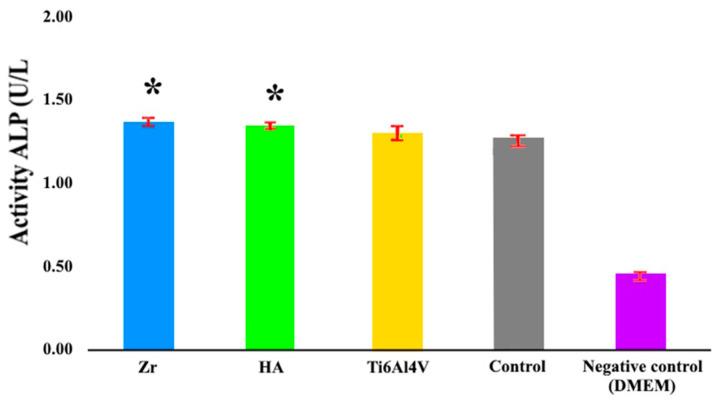
Alkaline phosphatase activity assay following 14-day osteogenic induction. Mesenchymal stem cells were cultured for two weeks in an osteogenic medium conditioned by implant materials. Osteogenic differentiation was assessed using the Alkaline Phosphatase Assay Kit (ab83369, Abcam). * *p* ≤ 0.05 compared to the control group.

Early osteogenic activity studies using alkaline phosphatase ELISA, performed after two weeks of differentiation, showed a statistically significant increase in alkaline phosphatase activity of 9% and 6% for the Zr and HA coating, respectively, compared to the Ti6Al4V alloy control group (Figure 11).

### 3.1. Antibacterial Assay

**Figure 12:**
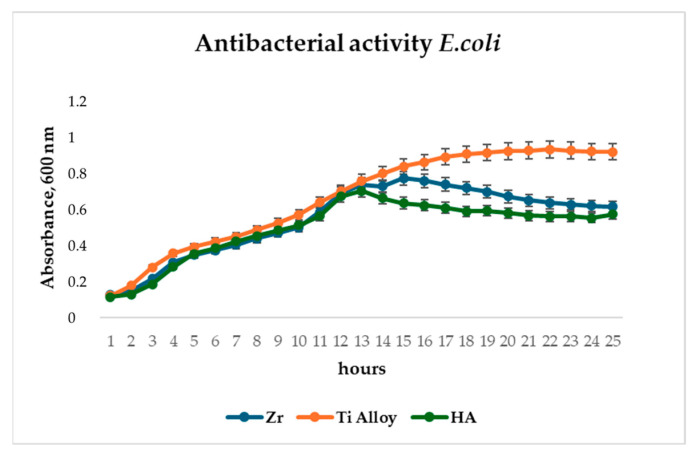
Antibacterial assay of Zr and HA coatings and Ti alloy on *E. coli*.

To evaluate the antibacterial potential of the microplasma-sprayed coatings, *E. coli* proliferation was monitored over a 24-h period in the presence of uncoated titanium alloy and HA-coated surfaces. The initial phases of bacterial growth, including the lag and exponential stages (up to approximately 12 h), proceeded similarly on both surfaces, as evidenced by parallel increases in OD600 values (Figure 12). However, a marked divergence in growth behavior emerged beyond the 12-hour mark. While bacterial populations on the Ti alloy surface continued to expand and eventually stabilized at an OD600 of around 1.0, cultures in contact with HA- and Zr-coated surfaces exhibited a delayed growth curve and plateaued at a significantly lower OD600 of approximately 0.6. This attenuation in absorbance suggests a reduction in bacterial viability or metabolic activity in the presence of the HA- and Zr-coating. Antibacterial analysis demonstrated 35% inhibition of *E. coli* after 14 h, reducing implant-associated infection risks (Figure 12).

### 3.2. Evaluation of the Morphological Features of MSCs When Attached to a Surface Material Using SEM

**Figure 13:**
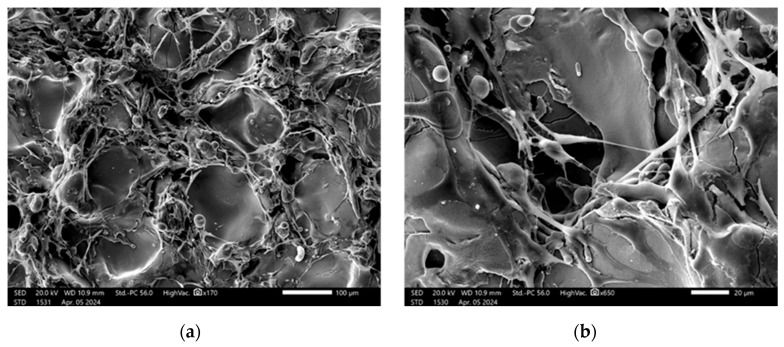

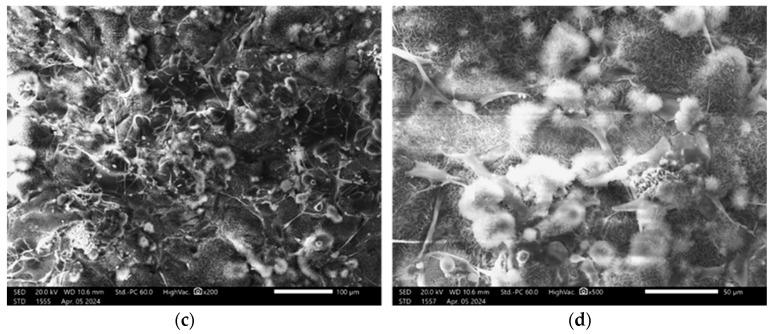
SEM images showing MSCs on the implant surface: (**a**,**b**) Zr-coated; (**c**,**d**) HA-coated. Scale bars are 100 µm (**a**,**c**), 20 µm (**b**), and 50 µm (**d**).

Cell attachment to the discs after two days of culture was assessed by SEM (Figure 13).

SEM analysis revealed that after two days of culture mesenchymal stem cells exhibited adhesion in the disc pores, displaying spindle-shaped fibroblast-like morphology characteristic of MSCs.

## 4. Discussion

Metallic or ceramic porous coatings are commonly applied to orthopedic implants to promote bone ingrowth, providing excellent fixation without the use of bone cement. Surface topography is known to play a critical role in osseointegration, and cellular response can be modulated by adapting the surface texture of the implant. Rough micro-, submicro- and nanoscale surfaces can be very effective in promoting osseointegration and can promote rapid bone growth and the formation of bonds between bone tissue and the implant surface [40]. In particular, for orthopedic titanium implants, the average surface roughness (Ra) recommended by a number of researchers is in a wide range from 0.07 µm to 100 µm [41,42]. Borsari, having studied the effect of roughness on cell proliferation, confirmed that surface morphology significantly affects cell behavior, but according to their data, excessively high roughness (more than 40 μm) leads to a decrease in cell proliferation [43].

As can be seen from the comparisons in Figure 3 and Figure 4, the roughness of the MPS Zr coating on the titanium alloy substrate after gas-abrasive machining is determined mainly by the MPS parameters, whereas the roughness of the HA coating also depends on the roughness of the substrate. The Zr–HA coating showed roughness higher than usually reported in the literature for HA coatings (1–8 μm) due to the presence of a Zr sublayer [44,45]. At the same time, Saeed Saber-Samandari et al. reported roughness of HA coatings up to 22.5 ± 1.1 μm [46].

As illustrated in Figure 5, the coatings exhibit surface pores measuring up to 100 μm in diameter. The formation mechanism for coatings with high surface roughness and porosity was previously detailed in our earlier work [32], where we demonstrated that such coatings result from the deposition of large, solid, heated particles traveling at low velocities toward the substrate. Due to their low impact speed, these particles undergo minimal deformation upon contact. Additionally, the particle size and their degree of heating within the microplasma jet can be controlled by adjusting the technological parameters of the microplasma spraying process.

The results of the microstructure analysis of the two-layer coating (Figure 6) show the presence of pores with a size of up to 250 μm in the Zr intermediate layer; the porosity of the Zr layer reached values of up to 20.3%, while in the HA upper layer the porosity reached 10%, and the pore sizes were up to 50 μm. According to modern scientific data, micropores form an environment favorable for osteoinduction—a biological process preceding osteogenesis. In contrast, macropores with developed vascularization contribute to the direct formation of bone tissue, bypassing the stage of chondrogenesis.

The results of numerous studies indicate that the minimum pore size required to stimulate bone ingrowth is about 100–150 μm [4,5,10,25,47]. This limit is determined by the morphological parameters of osteoclasts, the cells responsible for bone resorption. Pores that are too narrow can be blocked by individual cells, which limits the migration of other cells and complicates the diffusion of nutrients and the removal of metabolic products, disrupting cellular activity. From the point of view of ensuring adhesion, migration, and proliferation of osteoblasts, pores with a diameter of 200 to 400 μm are considered optimal. It is known that pores larger than 300 μm significantly contribute not only to the formation of new bone tissue but also to the development of a capillary network, which, due to improved vascularization, significantly enhances the processes of osteogenesis [47].

In addition, an increase in porosity contributes to a decrease in the elastic modulus of the implant material. This aspect is of particular importance for preventing aseptic loosening caused by the “stress shielding” effect, a phenomenon in which a mismatch between the elastic modulus of the implant and the bone leads to a decrease in the mechanical load on adjacent bone areas, which in turn causes a decrease in its density and a weakening of the bone structure. The effect of porosity on the elastic modulus is also confirmed in the work of Voinarovych et al. [48], where a direct relationship was established between the porosity of microplasma coatings made of Ti and Zr and their elastic properties. However, an increase in porosity is accompanied by a decrease in other mechanical parameters of the coating, especially the strength of its adhesion to the substrate. Therefore, optimization of the elastic modulus by increasing porosity is possible only up to a certain limit value, exceeding which can lead to a loss of implant stability due to insufficient adhesive strength of the coating compared to the strength of bone tissue.

The choice of the optimal thickness of the biocompatible coating is determined by the need to form a given porosity and surface topography that contribute to an increase in the contact area with bone tissue. A porous zirconium coating applied by microplasma spraying and having pores up to 300 μm in size meets the requirements for bone integration. Such a coating, according to our data (see Figure 6, Figure 7, Figure 8 and Figure 9), should have sufficient thickness—about 250–350 μm, while providing the necessary biomechanical and biological efficiency.

The average static peel strength of the Zr coating was 27.9 ± 3.8 MPa, while according to the requirements of ISO 13179-1:2021 for plasma-sprayed unalloyed titanium coatings on metal surgical implants, this value should be at least 22 MPa [49].

The determined average static peel strength of the HA coating on the Zr sublayer was 28.0 ± 4.2 MPa, which is almost two times higher than the minimum value of 15 MPa recommended by the ISO 13779-2 [50]. Taking into account the close values of the peel adhesion strength for the Zr coating and the two-layer Zr–HA coating, it can be assumed that the adhesion strength of the two-layer coating is determined by the adhesion strength of the thicker Zr sublayer.

Our hypothesis posits that the two-layer coating system provides synergistic functionality: the upper hydroxyapatite-based layer enhances osteoconductivity and supports cellular integration, while the intermediate Zr layer improves coating adhesion to the metallic substrate and enhances corrosion resistance, thereby contributing to long-term implant stability [51].

The results of cell proliferation study (Figure 10) allow us to confidently state that HA coatings create a more favorable environment for cell growth, which is an essential aspect for successful osseointegration of implants [52]. This indicates that although Ti6Al4V alloy is effective in supporting cell proliferation, new coatings may prove to be potentially more effective. The results obtained from the new coating are crucial for the superior efficiency of supporting cell growth, which is important in the context of implantology and regenerative medicine and further research. A material’s capacity to promote cell proliferation is essential for its effectiveness in implant applications, as it directly influences tissue healing and the successful integration of the implant with surrounding host tissue. The results show that the HA coating not only matches but also exceeds the performance of gas-abrasive-treated Ti6Al4V alloy in this regard. This implies the potential for the microplasma-sprayed HA coating to improve the outcomes of implant surgeries by providing better integration with the host tissue and promoting faster healing and recovery. The high viability of the coatings may also be related to the higher surface roughness and support area ratio, which characterize the level of surface expansion.

Assessing cell behavior during long periods of contact with an implant is vital to comprehensively understand its long-term biocompatibility and potential impact on tissue integration and healing processes [53]. In this context, the assessment of osteogenic capacity is particularly useful as it provides important data on the ability of the implant to promote successful osseointegration. The 9% and 6% increases in ALP activity (Figure 11) are significant in the context of bone healing and regeneration. These indicate that the coatings have a positive effect on early osteogenesis by enhancing the activity of osteoblasts, which are bone-forming cells. The importance of this finding lies in the potential of zirconia and hydroxyapatite as biocompatible materials to improve bone healing and regeneration. Hydroxyapatite is known for its biocompatibility and similarity to natural bone minerals, making it a popular choice for orthopedic and dental applications.

The elevated alkaline phosphatase activity observed in the HA group indicates that this material may create a more supportive environment for early-stage bone formation compared to the untreated control. This enhancement is especially significant in implantology and bone tissue engineering, where prompt and efficient osteogenesis plays a crucial role in the successful integration of implants and bone grafts. The results show that zirconia and hydroxyapatite coatings can improve the integration of implants with the surrounding bone tissue, potentially leading to improved outcomes in bone repair and regeneration procedures.

The observed antibacterial effect of HA and Zr coatings (Figure 12) may be attributed to the physicochemical properties of hydroxyapatite, including its surface roughness, ion release capacity, and electrostatic interactions, which may interfere with bacterial adhesion and colonization. These results support the hypothesis that HA and Zr coatings, in addition to their osteoconductive function, offer a degree of antimicrobial protection—an important consideration for the development of infection-resistant implant surfaces.

The process by which cells adhere to bone graft materials remains an area of active research. Scanning electron microscopy (SEM) is widely regarded as the most effective technique for observing cell behavior on material surfaces. Prior studies [54,55,56] have shown how bone-like cells interact with titanium surfaces during attachment. These cells are capable of sensing and responding to the three-dimensional features of the substrate due to their adaptable cytoskeletal structure. As such, variations in surface roughness can influence the strength of cell adhesion, which in turn affects key cellular functions including proliferation, migration, and differentiation [57]. The images presented in Figure 13 illustrate surface roughness as a key feature of the implant coatings, characterized by varying pore sizes and distinct topographic patterns. The rough surface morphology observed in the SEM images appears to play a significant role in facilitating cell attachment and proliferation. Consistent with previous studies that emphasize the role of surface roughness in modulating cellular behavior, our findings further underscore its importance in enhancing biological responses at the implant interface and promoting successful osseointegration [58,59].

SEM analysis offers valuable insights into the complex interplay between implant surface properties and cellular behavior, emphasizing the critical role of surface roughness and pore architecture in facilitating osseointegration. These findings carry significant implications for the design and optimization of implant surfaces aimed at enhancing biocompatibility and long-term clinical performance. The presence of filopodia and/or lamellipodia on the coated surfaces indicates active cell adhesion, spreading, and early stages of proliferation. Importantly, no signs of cytotoxicity were observed on any of the tested surfaces, suggesting that the sprayed coatings enhance the biocompatibility of titanium-based alloys and support favorable cell–material interactions.

These findings support the use of microplasma-sprayed HA–Zr bilayer coatings as a promising approach for improving the mechanical and biological performance of orthopedic implants. The integration of a zirconium interlayer not only enhances coating adhesion to the titanium substrate but also contributes to structural integrity under physiological loading conditions. The improved osteoconductivity of the hydroxyapatite surface was evident in the increased mesenchymal stem cell proliferation and viability, suggesting a favorable environment for early-stage osteointegration. Overall, the combination of mechanical robustness and enhanced cell compatibility suggests microplasma-sprayed HA–Zr bilayer coatings as a next-generation solution for advanced orthopedic applications.

## 5. Conclusions

This study demonstrated the effectiveness of MPS in producing biocompatible metal–ceramic bilayer coatings composed of a Zr sublayer and a HA top layer, intended for use on titanium alloy implant surfaces. The application of the Zr layer resulted in a significant increase in surface roughness (Ra = 24.72 ± 3.09 μm), while the addition of the HA top layer slightly reduced this value to Ra = 19.46 ± 2.29 μm—still notably higher than typical HA coating roughness values reported in the literature (1–8 μm). Such roughness is advantageous for promoting osseointegration.

Adhesion testing confirmed that both the single-layer Zr coating (27.9 ± 3.8 MPa) and the bilayer Zr–HA coating (28.0 ± 4.2 MPa) exhibit peel strengths exceeding the minimum thresholds indicating excellent mechanical integration with the substrate.

Biological assays revealed that coating composition had a pronounced influence on MSCs behavior. The HA-coated samples enhanced cell proliferation by approximately 45% within three days compared to untreated titanium alloy controls, which showed minimal effect on cell growth. These results underline the critical role of surface chemistry and topography in modulating cellular responses.

Altogether, the study confirms that MPS parameters can be strategically tuned to optimize surface characteristics, mechanical adhesion, and biological performance. The insights gained into the relationship between coating microstructure and its functional properties support the advancement of MPS-based technologies for the fabrication of high-performance, bioactive implant coatings. These findings offer a promising foundation for developing next-generation orthopedic and dental implants with improved osseointegration and long-term clinical outcomes.

## Figures and Tables

**Table 1:** Chemical composition of Zr wire brand KTC-125-705 (UNS R6070) [30].

Grade	Chemical Composition, %								
KTC-125	Hf	Fe	Ca	O	Si	Ni	C	Cr	Nb
	0.01	0.05	0.03	0.11–0.14	0.02	0.02	0.02	0.02	2.4–2.7

**Table 2:** Chemical composition of Ti6Al4V alloy.

Materials Grade	Ref.	Al	V	O	C	N	H	Fe	Ti
Ti6Al4V alloy	[33]	5.50–6.75	3.50–4.50	0.13	0.08	0.05	0.015	0.25	Bal.

**Table 3:** Parameters of gas-abrasive processing mode for sample surfaces.

Compressed air pressure, MPa	From 0.5 to 0.6
Distance from nozzle exit to work surface, mm	From 80 to 100
Angle of attack of the treated surface, deg	90
Linear speed of gun movement, mm/min	From 250 to 600
Processing time, min	From 0.10 to 0.12

**Table 4:** Chemical composition of Zr–HA coating assessed with EDX (Figure 6b).

	All Results in Weight%														
SP	O	Na	Al	Si	P	Cl	K	Ca	Ti	V	Fe	Ni	Sr	Nb	Zr
SP 1 (HA coating)	48.98				18.05			32.97							
SP 2 (Zr coating)	5.41												1.22	5.08	88.29
SP 3 (Zr coating)	4.47													4.35	91.18
SP 4 (Substrate Ti6Al4V)			3.78						94.03	2.2					

## Data Availability

The original contributions presented in the study are included in the article, further inquiries can be directed to the corresponding author.

## Abbreviations

The following abbreviations are used in this manuscript:

ALP	alkaline phosphatase activity
Cp-Ti	commercially pure titanium
EDX	energy dispersive X-ray analysis
ELISA	enzyme-linked immunosorbent assay
HA	hydroxyapatite
MPS	microplasma spraying
MSC	mesenchymal stem cell
Ti	titanium
SEM	scanning electron microscopy
Zr	zirconium
